# Advances in Cancer Therapeutics: Conventional Thermal Therapy to Nanotechnology-Based Photothermal Therapy

**DOI:** 10.3390/pharmaceutics13081174

**Published:** 2021-07-30

**Authors:** Sangeeta Kumari, Nilesh Sharma, Shivendra V. Sahi

**Affiliations:** 1 Department of Biological Sciences, University of the Sciences, Philadelphia, PA 19104-4495, USA; 2 Department of Biology, Western Kentucky University, 1906 College Heights Boulevard, Bowling Green, KY 42101-1080, USA; nilesh.sharma@wku.edu

**Keywords:** cancer, photothermal therapy, laser, clinical trial, magnetic nanoparticles, graphene, nanotubes, quantum dots, nanosheets, nanomaterials

## Abstract

In this review, advancement in cancer therapy that shows a transition from conventional thermal therapies to laser-based photothermal therapies is discussed. Laser-based photothermal therapies are gaining popularity in cancer therapeutics due to their overall outcomes. In photothermal therapy, light is converted into heat to destruct the various types of cancerous growth. The role of nanoparticles as a photothermal agent is emphasized in this review article. Magnetic, as well as non-magnetic, nanoparticles have been effectively used in the photothermal-based cancer therapies. The discussion includes a critical appraisal of in vitro and in vivo, as well as the latest clinical studies completed in this area. Plausible evidence suggests that photothermal therapy is a promising avenue in the treatment of cancer.

## 1. Introduction

Globally, cancer is one of the leading healthcare problems. It has caused enormous mortality in recent decades. Worldwide cancer incidence and mortality rates are continuously increasing. It is the second leading cause of mortality in humans [1]. Statistics indicate that it claimed 0.6 million human lives in the USA in 2019 alone [2]. During the last two decades, scientists have conducted extensive research in the field of cancer, which subsequently increased the knowledge in cancer biology [3]. It has been clearly shown that cancer cells have properties of metastasis, invasion activation, and evasion to growth suppressors, proliferative sustainability, cell death resistance, and angiogenesis. These properties make cancer treatment a challenging task [4]. Presently, surgery, radiation therapy, and chemotherapy, in variable combinations, are widely used for the treatment of cancer. One of the major drawbacks of conventional therapies is the cytotoxicity of healthy cells and a plethora of undesirable side effects inflicted to the patients [5]. This review article mainly focuses on the advances in radiation therapies that are transitioning from the conventional thermal therapy to laser-based photothermal therapy.

## 2. Thermal Therapies

**Figure 1:**
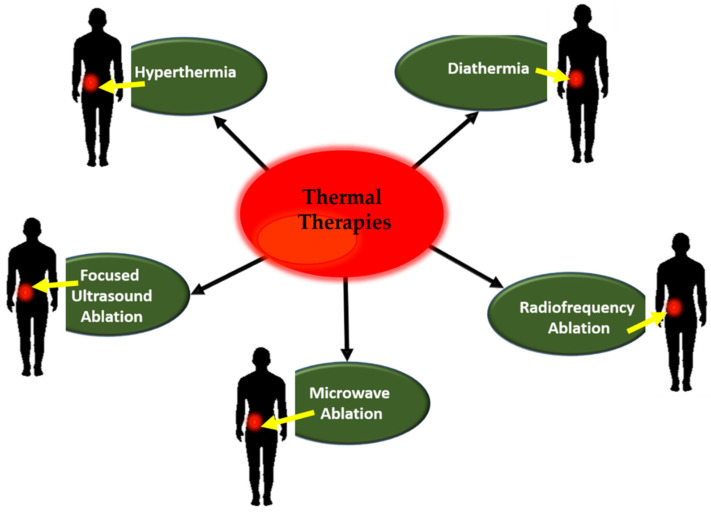
Types of thermal therapies.

In a biological system, temperature is considered as one of the most vital parameters for determining its viability and functionality [6,7,8,9]. Although elevated body temperature is not very beneficial for the human body and higher body temperatures have adverse effects on human health [10,11], it may be a boon for cancer patients [12,13]. Thermal therapies play an important function in treatments of cancer and were extensively used in the last quarter of the 20th century [12,13,14,15]. In recent years, interest in thermal therapies widened significantly, resulting in a plethora of peer-reviewed publications [16,17,18]. Figure 1 illustrates the various forms of thermal therapy applications.

### 2.1. Principle and Mechanism of Thermal Therapy

Thermal therapy is a technique for localized and controlled heating [19]. The basic principle of thermal therapy states that when the heat or cold is applied to the tissue, such as soft tissue, cutaneous tissue, or intra-articular tissue, it changes the core temperature of the tissue [20]. This change in core temperature of tissue leads to killing of the cells and modification of tissue [21,22]. Despite extensive research in the methods of thermal therapy, the exact mechanism of the effect of raised temperatures in single cells is not well understood. Host effect is one of the main reasons for this complexity. In thermal therapies, the temperature of a targeted tissue is raised for a certain period; this increase in temperature also induces thermal effects in the surrounding tissues [23]. The efficacy of thermal therapy depends on the duration of temperature treatment and the range of temperatures applied [23]. In thermal therapy, temperature of the part of the body or full body is raised above the body temperature for the duration of a few minutes to hours, which leads to death of the malignant tissue [24]. Based on these two factors, thermal therapies can be classified into hyperthermia, diathermia, and thermal ablation.

### 2.2. Hyperthermia

Hyperthermia is one of the well-known technologies in the field of cancer therapeutics [16,17]. In hyperthermia, the temperature ranges from 41 to 48 °C [25]. This therapy can be either given alone or it can be given along with other therapies, such as immunotherapy or surgical treatment, radiotherapy, and chemotherapy [12,26]. It has been observed that a combination of therapies is very promising, e.g., radiotherapy with hyperthermia [27,28,29,30,31]. The underlying mechanism of action in hyperthermia includes the denaturation of cellular protein when the tumor cells are exposed to a higher temperature, i.e., above 41 °C [32,33]. The denatured protein in the tumor cell aggregates and causes the destructive effects on tumor cells [34]. The increase in temperature also leads to an inactivation of tumor cells for a certain duration of time. Based on the area of application of heat, hyperthermia can be further classified in various categories.

#### 2.2.1. Local Hyperthermia

This strategy involves the application of heat to a confined focused area of the tumor, which leads to the death of cancer cells and nearby surrounding blood vessels. The effectiveness of this strategy depends on the high temperature and exposure time. This technique works by either applying heat to the body surface near the tumor externally with a machine or exposing the tumor to heat internally with a thin needle or probe [35]. The internal technique involves the insertion of a needle or probe in the tumor and the tip of the needle or probe releases energy, which results in overheating of the surrounding tissue and killing the tumor cells [28,36].

#### 2.2.2. Regional Hyperthermia

Regional hyperthermia is complex compared to local hyperthermia. In this strategy, a part of the body is heated, such as limbs, body cavity, or organs. This strategy is mainly used along with other cancer therapeutic techniques, such as radiation therapy or chemotherapy [37]. This regional hyperthermia is further classified as regional perfusion and hyperthermic intraperitoneal chemotherapy.

- Regional perfusion

In the human body, there are many parts where the blood supply is not typical of that of the rest of the body blood circulation. When the heating device is inserted into one of these portions, the blood from this part enters the heating device and then passes back into the original area to heat that particular area. It is a well-known technique in the field of treatment of melanomas and sarcomas.

- Hyperthermic intraperitoneal chemotherapy

This technique is a well-known technique for the treatment of peritoneum cancer. In this technique, heated chemotherapy drug is circulated during the surgery through the peritoneum cavity.

#### 2.2.3. Whole Body Hyperthermia

This strategy is more complex than local and regional hyperthermia. This has been predominantly applied to metastatic cancers. In this method, the temperature of the human body is raised by various strategies, including thermal chamber, warm water immersion, and heating blankets [38]. The temperature of the human body is raised up to 43 °C. It has been reported that, following the rise in body temperature, certain immune cells in the human body become active for a certain time, which leads to an increase in cell-killing components circulating through the blood.

### 2.3. Diathermia

Diathermia is a mild heat treatment. In this treatment, cells are subjected to the temperature of up to 41 °C. This temperature range does not cause many changes at the cellular levels, but it induces beneficial functions, such as blood flow increase, pH change, physiological changes, and oxygenation [39]. Due to its beneficial properties, this technique is commonly used in physiotherapy.

### 2.4. Other Conventional Thermal Ablation Techniques

Thermal therapies are well-known techniques used in the treatment of various types of cancer [12,13]. In these therapies, the temperature of the target cancer tissue is raised for a given time. There are many thermal ablation techniques used currently.

#### 2.4.1. Radiofrequency Ablation

Radiofrequency ablation (RFA) is a well-known technique used for the ablation of cancer in patients where surgical procedures are not possible [40,41]. It has been used for the ablation of cancers such as bone, brain, lung, breast, pancreas, and kidney [42,43,44]. In RFA, a high-frequency ablation probe is used for the ablation of a target cancer tissue. The radio frequency used for this technique is above 375 kHz up to 500 kHz. In the RFA technique, the heat produced by the probe in the surrounding cancer tissue increases the temperature, ultimately killing the cancer tissue [45]. The probe used in this technique could be needle-like, bipolar, or elliptical [46,47,48,49,50,51,52]. Due to its excellent outcome, it has received a lot of attention from scientific communities.

#### 2.4.2. Microwave Ablation

The microwave ablation (MWA) technique is more promising than the RFA technique. One of the reasons is that a wide area could be heated with the help of multiple applicators with the MWA technique. The MWA technique is widely used in the field of radiology to cure cancer. The basic principle of heating caused by microwave is, when a body part is exposed to the electromagnetic wave, the water molecules in the exposed body part begin to align in the direction of the applied field, leading to a heightened energy level that increases the body temperature [53]. In this technique, electromagnetic waves within the frequency range 915–2.45 MHz are applied [41,54]. This frequency of electromagnetic waves through the applicator generates heating in tumor tissue. The heat generation from the applicator is very uniform. The heating produced by the MWA is deep; therefore, extra precaution should be taken while using this technique to avoid any serious damage to deep normal tissues.

#### 2.4.3. Focused Ultrasound Ablation

Focused ultrasound ablation is also known as ultrasound ablation or high intensity focused ultrasound ablation (HIFU). This technique is very promising for cancer ablation. It provides a selective target for the cancer tissue. It can heat the selective tissue up to the temperature of 100 °C within 10 s. This is an important technique for targeting deep tumors. The underlying mechanism includes the effect of an impinging ultrasound beam, which leads to heating of the tissue and killing the cancer cells by necrosis [53,55,56,57]. Based on the application root, this technique can be further classified as extracorporeal (treat superficial tumor), transrectal (treat prostate cancer), interstitial (deep tumors), or percutaneous (very deep tumors) [58,59,60].

### 2.5. Role of Nanotechnology in Thermal Therapies

Nanotechnology has an important impact on thermal-therapy-based cancer therapeutics. It has demonstrated vital applications in diagnostics and treatments [61,62,63,64]. It is observed that nanoparticles-based thermal therapies have many advantages over conventional cancer therapy methods. Nanoparticles are known to have unique optical and magnetic properties. Due to these properties, it is observed that nanoparticles can raise the temperature in cancer cells by absorbing radiofrequency waves (RW), electromagnetic waves (EW), and near infrared light (NIR) [65,66]. Nanoparticles can cause localized and remote heating of the tumor cells. The advantage of using nanoparticles for thermal therapies in a biological system is due to their small size, biocompatibility, dispersibility in biocompatible solvent, bioavailability, and they are capable of producing heat when excited externally [67,68,69]. Various types of nanoparticles are reported in the application of thermal therapy. They can be broadly categorized into magnetic [70] and non-magnetic nanoparticles [71].

#### 2.5.1. Magnetic Nanoparticles Dependent Thermal Therapy: Mechanism of Action

Magnetic nanoparticles have an important role in thermal therapies. In this method, the magnetic nanoparticles are introduced into the tumor cells. The release of nanoparticles to the targeted site can be achieved by either direct injection or by targeted delivery to the tumor. Once the magnetic nanoparticles in tumor cells are subjected to an external alternating magnetic field of optimum frequency and intensity [72,73,74], it induces heating of the magnetic nanoparticles, which successively leads to heating of the tumor cells and their destruction. The mechanism that causes the heating of the magnetic nanoparticle upon exposure to the alternating magnetic nanoparticle include brown relaxation, N’eel relaxation time, and hysteresis loss [75,76,77,78,79]. Upon heating of magnetic nanoparticles in tumor cells, the heat is immediately conducted to the surrounding tissue, and the temperature is maintained at 42 °C for 30 min, causing the annihilation of tumor cells [75,76]. These magnetic nanoparticles with heating effects have also found applications in tumor imaging and as an MRI contrast agent [73,79]. One of the well-known examples of a magnetic nanoparticle is iron oxide nanoparticle Fe_3_O_4_.

##### Iron Oxide Nanoparticles

Iron oxide nanoparticles are widely used in biomedical fields. These nanoparticles are biocompatible, biodegradable, nontoxic, and have unique magnetic properties [80,81,82,83,84,85]. Fe_3_O_4_ nanoparticles have extensive applications in magnetic resonance imaging (MRI) as a contrast agent. It has shown promising applications in the field of cell targeting and drug delivery. Cell targeting from Fe_3_O_4_ nanoparticles is successfully achieved with targeting molecules due to the unique surface area of Fe_3_O_4_ nanoparticles [86]. As discussed above, these Fe_3_O_4_ nanoparticles in the tumor tissue cause magnetic hyperthermia when subjected to an alternating magnetic field, resulting in heat production, which eventually kills the tumor tissue [87]. It has been observed that the magnetic hyperthermia induced by an alternating magnetic field may also cause damage to healthy tissues surrounding a tumor. This effect can be reduced by using NIR laser radiation. NIR laser radiation is safer for healthy cells than alternating magnetic fields [88].

Although magnetic nanoparticles have shown promising results in thermal therapy, non-magnetic nanoparticles, such as metal nanoparticles, nano shells, and nanotubes, are being explored for cancer thermal therapy. One of the latest technologies, which is based on heat treatment, uses photothermal therapy.

### 2.6. Photothermal Therapy by Nanoparticles: Mechanism of Action

In photothermal therapy, a laser light of definite power and wavelength is used for killing cancer cells [75,76,89,90]. In this method, when cancer cells carrying nanoparticles inside or surrounded by nanoparticles are exposed to a definite laser, the laser beam causes oscillation of the nanoparticles, resulting in heating of these particles. Exposure of laser light to nanoparticles causes excitation of electrons in the nanoparticles, followed by a non-radiative relaxation of electrons. The relaxation of electrons leads to kinetic energy gain, which will result in heat induction in nanoparticles [91,92,93]. The heat from nanoparticles is conducted in cancer tissue, which leads to the destruction of cancer cells. Despite the success of photothermal therapy, there are some limitations to laser-based photothermal therapy; sometimes it is not capable of providing adequate heat to the tissue [94,95,96].

### 2.7. Photothermal Therapy by Laser Applicator: Mechanism of Action

The factors that are making photothermal therapy a promising technology in cancer therapeutics include the use of a laser and the medium of transport of the laser in cancer tissues. The mechanism of photothermal therapy involves a laser of definite laser energy, its power, exposure time, wavelength with appropriate applicator, and physical properties of cancer tissues [97]. These applicators are mainly thin optical fibers, which induce the laser light inside the tumor tissue [98]. The laser light induced is well absorbed by the tumor tissue, leading to heating of the tissue. When the temperature of the tumor tissue reaches up to the temperature range of 45–55 °C and is retained for a period, it leads to irreversible damage in the cancer tissue [98]. Arrhenius rate analysis (a mathematical description) denotes the cell death as a function of exposure time of laser and temperature [99].

One of the major concerns to any of the photothermal therapies is how to reduce the effect of treatment on normal healthy tissues surrounding the tumor. This can be achieved by keeping the factors in consideration, such as optimized laser setting, laser applicator, tumor tissue temperature, and physical and optical properties of the tissue [100]. For treating the superficial tumor, an optimum depth of the applicator of the laser is needed. A deep optical penetration depth of the laser is required to destroy deep cancer tissue.

## 3. Laser Technology for Photothermal Therapy

Laser-based cancer tissue ablation is one of the well-known technologies in cancer therapeutics, although, there are many other technologies known for cancer thermal therapy, such as cryosurgery, microwave, high intensity ultrasound, and radio frequency, which have also shown promising outcomes in cancer therapeutics. However, laser-based thermal therapy, known as photothermal therapy, is a well-known technology used in the application of cancer therapeutics [101].

Clinical and preclinical trials for the use of a laser in different cancer tissues started during the period of the 1980s. Application of this technology allows destruction of various types of cancers, including brain cancer, liver cancer, pancreatic cancer, and prostate cancer [102,103].

### Type of Lasers

Laser settings play vital roles in photothermal therapy. Photothermal therapy can be performed with two types of laser settings: pulse mode laser setting and continuous mode laser setting. In pulse mode, the laser is released from its source in a non-uniform manner. Due to this reason, a high-power laser is used, normally >100 W, which will raise the tumor tissue temperature and lead to irreversible damage to the tumor tissue [104]. In continuous mode, the laser is exposed to the tumor tissue uninterrupted. If the laser power is low, then the laser is exposed for a longer time. If the laser power is high, the exposure time for tissue is reduced [105].

**Figure 2:**
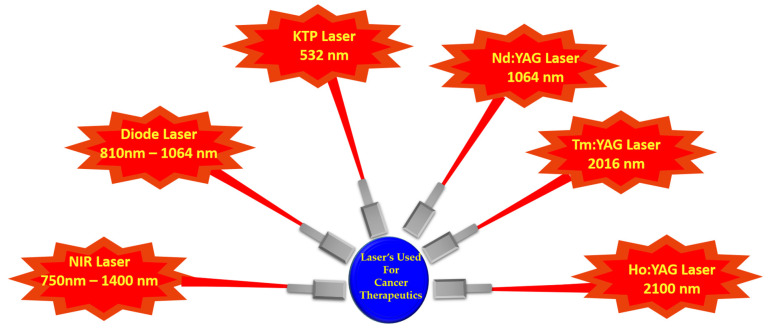
Types of lasers used for cancer therapeutics.

Various types of lasers have been used in photothermal therapy for cancer treatment (Figure 2). These lasers differ from one another only in the absorption length and wavelength. Near infrared (NIR) laser is widely used in photothermal therapy due to its deep penetration effects. For KTP laser (Potassium titanyl phosphate), the wavelength is 532 nm; it is absorbed deeply by the blood cells containing hemoglobin. The penetration depth of this laser is more than 1000 nm. This laser can be used in either continuous mode or pulse mode. Diode laser is predominantly used in photothermal therapy. The wavelength range for this laser varies from 800 to 1000 nm. The penetration depth of this laser is 1 nm to 50 nm, depending upon the tissue to which it is applied. It can be used in both pulse and continuous modes. Nd:YAG laser (neodymium-doped yttrium aluminum garnet) is the most widely used laser in photothermal therapy. It is mostly used in a continuous mode. The wavelength of this laser lies in the range of 1064 nm. The depth of penetration is 1 to 10 cm [106]. Tm:YAG laser (Thulium yttrium aluminum garnet), with a 2016 nm wavelength range and penetration depth of 1 cm, is a suitable laser type. This laser can be used in a continuous mode. Ho:YAG laser (Holmium yttrium aluminum garnet), with a wavelength range of 2100 nm and penetration depth of 0.04 cm, can be used in the pulse mode.

## 4. Potential of Nanomaterials for Photothermal Therapy

**Figure 3:**
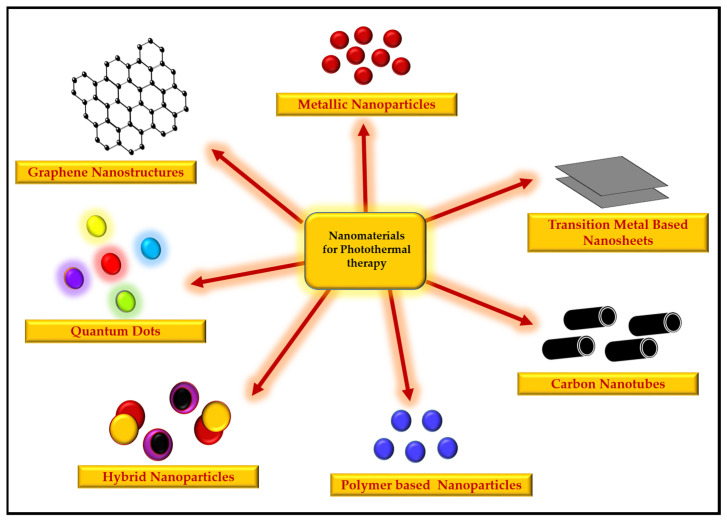
Types of nanomaterials used for photothermal therapy.

There are many types of nanomaterials that have been studied variously for photothermal therapies, and include metallic nanoparticles, organic nanoparticles, carbon nanotubes, hybrid nanoparticles, transition-metal-based nanosheets, quantum dots, and polymer-based nanoparticles (Figure 3, Table 1).

### 4.1. Metallic Nanoparticles

Metallic nanoparticles have predominant applications in the field of cancer nanomedicine. Among metallic nanoparticles, gold nanostructures were widely explored by researchers worldwide. It is well known that gold nanoparticles can be modified in nanostructures, such as nanorods, nanoshells, nanospheres, and nanocages, depending upon their synthesis procedures. Since the vascular system of tumor cells have wide gaps in their dispositions, varying from 100 nm to 2 µm, gold nanoparticles can easily penetrate the tumor cells. It has been reported that the tumor cells lack the lymphatic clearance mechanism, which allows the internalized gold nanoparticles to deposit inside the tumor cells [107].

Gold nanoparticles have excellent optical properties, which can be manipulated by modifying their morphologies. The gold nanoparticles of a complex morphology display an enhanced localized surface–plasmon resonance (LSPR) effect [108,109]. Manikandan et al. synthesized platinum nanoparticles and used these for photothermal therapy in neuro-2A cells [110]. Platinum-nanoparticle-internalized neuro-2A cells were then irradiated with an NIR laser of wavelength 1064 nm. This study indicated that these platinum nanoparticles raised the temperature of cells up to 9 °C, resulting in destruction of the cancer cells. It was inferred that platinum nanoparticles are an excellent tool for killing neuro cancer cells [110]. Tuan and Hu et al. synthesized polyethylene glycol (PEG)-coated copper nanowires. These PEG-coated copper nanowires were studied in vitro and in vivo. In vitro study indicated that these PEG-coated copper nanowires had twisted around the cancer cells. When the cancer cells were exposed to the 808 nm NIR laser, the temperature of the cancer cells increased to more than 50 °C in 6 min, causing cancer cell death. In vivo study was performed on mice having colon tumors. These PEG-coated copper nanowires were injected into the tumor, followed by the tumor’s exposure to an NIR laser of 808 nm for 6 min. NIR exposure to tumor raised the temperature of the tumor to over 50 °C, which finally led to significant tumor growth suppression and caused necrosis. Since the synthesis of these nanowires is cost effective, it can be considered as a viable option for photothermal treatments [111].

### 4.2. Graphene Nanostructures

In the recent years, graphene-based nanomaterials have attracted the attention of the scientific community due to their wide applications in biomedical fields, including drug delivery, biosensors, and photothermal therapy [112,113,114]. It is well known that graphene/graphene oxide nanosheets have superior physical and biological properties. Physical attributes involve thermal, electrical, and mechanical properties [115,116,117,118,119,120,121,122]. The NIR graphene exhibits remarkably high photon absorption, which renders it fit for the photothermal therapy. The ability of graphene/graphene oxide nanomaterials to cause local heating makes them a promising candidate for photothermal therapy of tumors [123,124,125,126,127,128]. Although graphene and its derivatives have wide applications, still the graphene is not completely biocompatible due to its poor solubility in biological fluids. For its application in photothermal therapy, it is important that the graphene-based heating agents should have good solubility in a physiological fluid. To achieve this, the surface of graphene is functionalized to minimize the limitation of biotoxicity [114,129,130]. The efficacy of heat production from graphene and graphene-oxide-based heating agents upon exposure to laser irradiation is determined and compared with gold nanoparticles by the factors of time constant and sphere method [131]. Liu et al. reported successful in vivo uptake of polyethylene-glycol-functionalized graphene oxide (GO-PEG) nanosheet in 4T1 murine breast cancer. The laser of low power 2 W cm^−2^ was used to irradiate the GO-PEG nanosheets, which caused the local ablation of cancer tissue. The synthesized GO-PEG nanosheets were labelled with fluorescent dye Cy7 by conjugation to visualize the localization of nanosheets in mice organs. Their results indicated that most of the functionalized nanosheets were accumulated in mice kidney and tumor. This study reported survival of all the treated mice [132]. Yang et al. synthesized gold-nanoparticle-specific aptamer–graphene oxide. This functionalized GO was used for targeted heat generation in MCF-7 human breast cancer cells, upon irradiation with NIR laser. The results indicated that GO was an excellent candidate for photothermal therapy of breast cancer [133].

### 4.3. Carbon Nanotubes

Carbon nanotubes (CNT) have been widely used in biological systems, which include development of biosensors, nanocarrier-based delivery in cells, biomedical devices, and bioelectrochemistry [134,135,136,137,138]. CNT have shown extensive optical absorbance in the NIR range [139,140,141]. Due to the excellent absorbance in the NIR range, significant heat is released by the CNT, which leads to thermal destruction of cancer cells [142]. Therefore, CNT is considered important for photothermal therapy. It has been reported that CNT, in general, induces necrosis and inflammation in biological systems, but this effect of CNT is reduced by oxidation of CNT [143]. Oxidation of CNT causes reduced length of CNT. Like graphene, CNT also have less solubility in aqueous medium. The solubility of CNT is enhanced by PEG-functionalization. Functionalization of CNT ultimately enhances its biocompatibility and penetration ability through the cell membrane [144,145,146]. Studies have demonstrated that the multiwalled CNT have greater potentials of generating heat from NIR radiations [147]. Zhou et al. synthesized folate (FA)-functionalized single-walled carbon nanotubes (SWNT). Folate binds to the biomarker of tumor cells. Since folate facilitates the targeted delivery of SWNT in tumor cells, the FA-SWNT was irradiated with a 980 nm laser. This study showed that FA-SWNT caused a significant destruction of targeted tumor cells in both in vitro and in vivo studies. Non-targeted healthy cells were not affected significantly by this treatment [148]. Sobhani et al. synthesized oxidized CNT (O-CNT), and further functionalized it with PEG. They observed that O-CNT had less toxicity compared to a multiwalled CNT. The PEG-functionalized O-CNT has shown surprisingly the least toxicity towards cancer cell lines, such as HepG2 and HeLa. However, the role of PEG-functionalized O-CNT was much different in the in vivo study that involved the illumination of the PEG-functionalized O-CNT with a continuous wave laser of wavelength 808 nm and power of 2 W cm^−2^ for the duration of 10 min. This study indicated a significant reduction in the tumor size. PEG-functionalized O-CNT also appears, thus, as a promising heating agent for photothermal therapy [139].

### 4.4. Quantum Dots

In recent years, quantum dots have been widely studied in research. These are nanosized crystals, which are popularly used in biomedical applications. They have shown a continuous and broad spectrum, high fluorescence quantum yield, and stability towards photobleaching [149,150,151]. Due to these properties, quantum dots are considered to be an efficient fluorescent probe for proteins and organic dyes. This quantum-dot-probe protein has an important application in in vivo and in vitro biomedical imaging [152,153]. Chu et al. reported the therapeutic efficacy of CdSe and CdTe quantum dots. Silica-coated CdTe quantum dots were injected into mouse melanoma tumor and irradiated with a laser of 671 nm. It was observed that the tumor growth was inhibited dramatically upon irradiation with the laser; the tumor completely vanished over a period of time [154]. Mohapatra et al. used a unique source of N-rich mesoporous carbon nanospheres that were uniform in size and had void spaces. These void spaces of the matrix capture the nitrogen-doped quantum dots (NCQD). These nanospheres exhibited efficient fluorescence quantum yields and light-to-heat conversion property. When human oral cancer cells (FaDu) were treated with these nanospheres, they caused a significant thermal ablation to the cancer cells upon irradiation with a 980 nm NIR laser [155].

### 4.5. Hybrid Nanoparticles

Hybrid nanoparticles are generally formed by combining two nanoparticles. These two nanoparticles form either core shell or nanocomposites. Hybrid nanoparticles are multifunctional with novel chemical and physical properties [156]. Hybrid nanoparticle synthesis leads to enhancement in their optical properties [157]. Khafaji et al., synthesized a Au–Fe_3_O_4_ hybrid nanostructure protected with PEG. They compared the heat generation potentials between an unprotected Au–Fe_3_O_4_ hybrid nanostructure and a protected Au–Fe_3_O_4_ hybrid nanostructure. Au–Fe_3_O_4_ hybrid nanoparticles, when transfected to human breast adenocarcinoma, the cancer cells internalized the Au–Fe_3_O_4_ hybrid nanoparticles. These carcinoma cells were further irradiated with NIR laser (wavelength 808 nm with power of 0.5 W cm^−2^) for the duration of 7 min. The observations of this study indicated that only 32% of the human breast adenocarcinoma cells could survive upon irradiation while 68% of cells were destroyed by laser irradiation [158]. Cai and Ding et al. used Au–Pd hybrid nanoparticles, functionalized with folic acid and chlorin e6. In this hybrid nanostructure, Au was found in the shape of an eyeball and palladium as a shell of the Au eyeball. The folic acid acts as a cell-targeting molecule and Ce6 acts as a photodynamic agent. Again, when these hybrid nanoparticles were transfected to MCF-7 breast cancer cells, the nanoparticles were well internalized by the cells, which, in turn, were irradiated with an NIR laser of 808 nm and power 1.5 W cm^−2^. Observations in this study also indicated that the hybrid nanoparticles were accumulated in the target tumor and were able to be well irradiated with the laser, leading to a significant damage of cancer cells. Au–Pd hybrid nanoparticles are proving to be an effective tool of ablation for breast cancer therapy [159].

### 4.6. Transition-Metal-Based Nanosheets

Transition-metal-based nanosheets have attracted the attention of the scientific community worldwide. These are also referred to as transition metal dichalcogenides (TMD). Besides being highly biocompatible, these nanosheets have incredibly unique and efficient photothermal efficacy and, thus, they are considered as a highly efficient photothermal agent. TMD also exhibits significantly strong absorption in the NIR region and spin orbit coupling, making it an ideal photoacoustic imaging contrast agent [160]. Wei et al. synthesized CoFeMn dichalcogenides (CFMS) nanosheets, which were further modified with polyvinyl pyrrolidone. It was observed that CFMS nanosheets had excellent chemo-dynamic properties and were effective agents in photoacoustic imaging and photothermal therapy. Results indicated that CFMS nanosheets were capable of removing a tumor completely in in vivo conditions. It also caused complete apoptosis in HepG2 cells [161]. One of the well-known nanosheets is MoS_2_ nanosheet. MoS_2_ nanosheets are known to have large surface areas that make them useful as a drug delivery agent, a photothermal agent, or a chemotherapeutic agent [162]. Feng et al. synthesized unique flower-like MoS_2_ nanoflakes, which were further modified with PEG. These nanoflakes were used for the study of photothermal effects in cancer cells. In this study, 4T1 cancer cells were transfected with MoS_2_-PEG nanoflakes, then subjected to an NIR laser of wavelength 808 nm, with different power ranges. After laser irradiation of cells at a power of 2 W cm^−2^ for 10 min, and after 24 h of incubation, 4T1 cell viability was recorded to be only 38.9% [163]. Wang et al. synthesized ammonium ion intercalated 1T-WS_2_ ultrathin nanosheets (N-WS_2_), which were used for in vitro and in vivo photothermal therapy studies. These nanosheets were tested on cell lines, including HeLa cells. The nanosheets internalized by the HeLa cells were irradiated with an NIR laser of 808 nm wavelength. Findings in this study indicated that these nanosheets had exhibited promising photothermal conversion efficiency in vitro, as well as in vivo conditions [164].

### 4.7. Polymer-Based Nanoparticles

Polymer-based nanoparticle is an advanced area in the field of NIR photothermal therapy. An important concern associated with inorganic nanoparticles is potential cytotoxicity due to the accumulation of inorganic nanoparticles. To overcome this issue, scientists have developed polymer-based nanoparticles. The polymer materials which are being investigated are polyaniline, polypyrrole, polydopamine, and poly-(3,4-ethylenedioxythiophene): poly (4-styrenesulfonate) (PEDOT: PSS). It is reported [165,166,167,168] that these polymer materials have shown promising photothermal effects. It is observed that these polymer-based nanoparticles are having enhanced biocompatibility and biodegradability [169]. Zhou et al., synthesized F-127 surfactant-functionalized polyaniline nanoparticles (F-PANP). The functionalization of nanoparticles with F-127 increases the hydrophilicity of nanoparticles. They tested the NIR laser photothermal effect of this nanoparticle in vitro in the HCT116 cancer cell line. The result indicated increased cell death. Further, they tested F-PANP in vivo in mice having HCT116 xenograft tumor. The tumor was irradiated with 808 nm of 0.5 W cm^−2^ NIR laser for the duration of 3 min, and a significant suppression in tumor was observed [170]. Wang et al. synthesized folic-acid-functionalized lipid-coated polyaniline nanoparticles. Folic acid is used to achieve the targeted delivery at the tumor site. They studied the NIR photothermal effect of these nanoparticles in vitro in HeLa cells, as well as in vivo in BALB/c mice expressing HeLa-induced tumor. The result indicated that NIR-laser-based photothermal therapy induces apoptosis and necrosis in tumor cells in vitro and in vivo [171]. Manivasagan et al. synthesized chitosan-polypyrrole-based nanocomposites and studied their NIR photothermal effect in vitro in MDA-MB-231 cells (breast cancer cell line). For in vivo assessment, they injected nanocomposite intratumorally in mice. The cells (in vitro) and tumor (in vivo) were irradiated at 808 nm (2.0 W cm^−2^, 5 min). In vitro results indicated cell death, while in vivo results showed significant reduction in tumor size [172]. Wang et al. synthesized polypyrrole hollow nanocapsules (PPy HNCs). They loaded doxorubicin hydrochloride (DOX) in PPy HNCs and studied the NIR-laser-based photothermal effect in BALB/c nude mice having HepG2 tumor [173]. Wu et al. synthesized polydopamine-coated cluster of iron oxide nanoparticles. They tested the photothermal effect of this nanoparticles in HeLa cells and HepG2 cells. In this case, nanoparticle-incubated cells were irradiated with NIR laser radiation for 10 min with the power of 2 W cm^−2^ [174]. Liu et al. used polyethylene glycol (PEG)-coated PEDOT:PSS nanoparticles as a drug carrier. In this study, anticancer drug doxorubicin (DOX), SN38, and photodynamic agent chlorin e6 (Ce6) were loaded to PEDOT:PSS-PEG separately. Further, they delivered these nanoparticles to cancer cells 4T1 and exposed these cells with a laser of appropriate wavelength, power, and duration. Dox-loaded PEDOT:PSS-PEG nanoparticle incubated in 4T1 cancer cells were irradiated with an NIR laser of 808 nm for 20 min at a power of 0.15 W cm^−2^ [175].

## 5. Effect of Nanoparticle Shapes and Sizes on Photothermal Therapy

In photothermal therapy, the shape and size of nanoparticles have an important impact. Nanoparticles have unique thermal and optical properties, which can be modified by tuning the surface chemistry, size, and shape of the nanoparticles [176]. This unique property of nanoparticles make them an excellent tool for photothermal therapy [177]. Gold nanoparticles have been shown to be one of the versatile candidates for photothermal therapy. Alteration in shape and size of the gold nanostructure greatly influences their optical properties. In photothermal therapy, when the light of definite wavelength falls on the surface of gold nanoparticles, it leads to the oscillation of surface electrons of the nanoparticles, known as localized surface plasmon (LSP). It is reported that gold nanoparticles exhibit extraordinarily strong scattering, as well as absorption of light when the electron oscillation frequency matches with incident light frequency [178,179,180,181,182]. Gold nanoparticles’ heating efficiency depends upon the wavelength of excited light and LSP resonance. It has been demonstrated that the complex-structured gold nanoparticles, such as AuNP with sharp or elongated ends, have higher heat generation capacity than the spherical gold nanoparticles. The reason for high heat generation in complex nanoparticles is due to more enhancement of laser light by the elongated or branched structures, which results in increased light absorption and, furthermore, heat generation [183,184,185]. The protrusion in the morphology of complex gold nanoparticles enhances their polarizability. In plasmon resonance wavelength, this enhancement in polarizability leads to a red shift [186]. The size of the gold nanoparticles also displays an important impact on photothermal therapy. Large-sized gold nanoparticles act as a better probe than small-sized gold nanoparticles in imaging-guided photothermal therapy. This is because a large-sized gold nanoparticle has better resolution than smaller ones. On the contrary, smaller gold nanoparticles have shown a better efficacy towards photothermal therapy than larger nanoparticles. Smaller nanoparticles need very low laser energy to show its effect, i.e., to kill the cancer cells. It is also reported that smaller gold nanoparticles (diameter < 5 nm) are eliminated from the body by renal clearance; however, large-sized particles are retained in the body for a longer time and their elimination rate is extremely low [187].

## 6. Application of Nanomaterials in Cancer Therapy Studies

Photothermal therapy is a well-known technology used in the application of cancer therapeutics. Scientists are investigating the efficacy of photothermal therapy in cancer research in in vitro, in vivo settings and in human clinical trials.

### 6.1. In Vitro Photothermal Therapy

**Figure 4:**
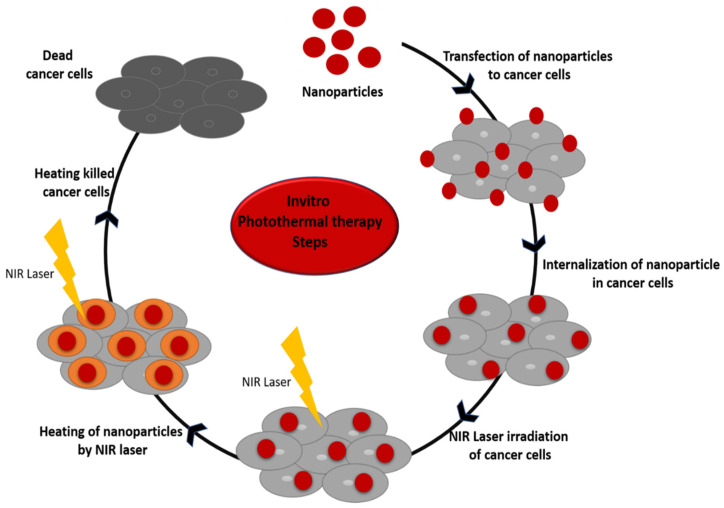
Steps for in vitro photothermal therapy.

In vitro photothermal therapy is the primary step in the testing of nanomaterials as a potential photothermal agent in the biological environment. In the conditions of in vitro testing, the nanoparticles are transfected to the cells and incubated at 37 °C for a certain duration of time. As discussed above, nanoparticles internalized by the cell are irradiated with a laser, which causes heating of the nanoparticles, leading to cell death (Figure 4). This suggest that the heating intensity of nanoparticles inside the cell can be regulated by the incubation time of cells with nanoparticles. It has been observed that the greater the incubation time of cells with nanoparticles, the greater the internalization of nanoparticles inside cells, which results in greater heat production by the nanoparticles upon laser irradiation. Thus, incubation time plays an important role in heat intensity production inside cells. Other factors that affect the internalization of nanoparticles include size, shape, and surface chemistry of nanoparticles. Song et al. developed carbon-coated MoO_2_ nanoparticles. MoO_2_ nanoparticles display strong photo-absorption in the NIR region. In in vitro MDA-MB-231 breast cancer cells, as well as in vivo, photothermal study of these nanoparticles indicated that they are excellent for photothermal therapy application [188]. Chen et al. conjugated gold nanoparticle with anti-mucin-7 antibody, which was used as a targeting molecule. The anti-mucin-7-antibody-conjugated gold nanoparticles were transfected to urothelial cancer cells. Once the nanoparticles were internalized by these cancer cells, they were exposed to a laser of 532 nm (green laser) at a power of 10 W cm^−2^ for 300 ms, which killed the cancer cells effectively. They also observed that, if the gold nanoparticles are not conjugated to the antibody, they need more power 30 W cm^−2^ and more time to show the same effect as antibody-conjugated nanoparticles [189]. Chen et al. studied the photothermal therapy effect of copper sulfide nanoparticles in cervical cancer HeLa cells. The nanoparticle-treated cells, when irradiated with a laser of 808 nm, caused a significant photothermal ablation of the cancer cells [190].

### 6.2. In Vivo Photothermal Therapy

**Figure 5:**
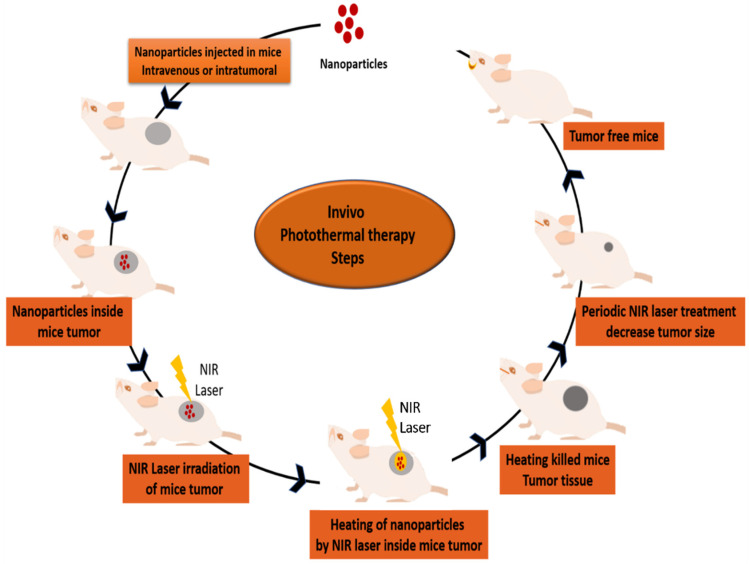
Steps for in vivo photothermal therapy.

In vivo photothermal therapy investigation is an important study in photothermal therapy research. In vivo photothermal effect depends mainly on the concentration of nanoparticles in a tumor. In an in vivo photothermal therapy study, nanoparticles (PTT agent) were injected into mice by two well-known ways: intratumorally or intravenously (Figure 5). Zheng et al. synthesized polypyrrole nanoparticle (PPYNPs). The mode of injection of PPYNPs was intravenous in mice. PPYNPs were observed to be a very promising photothermal agent for in vivo photothermal therapy. They have a strong absorption in the near infrared region and exhibit a photothermal efficiency of ∼45% at an NIR of wavelength 808 nm [191]. In another study, Cheng et al., synthesized a novel photothermal agent, branched PEG conjugated poly-(3,4-ethylenedioxythiophene):poly(4-styrenesulfonate) (PEDOT:PSS-PEG) [192]. This photothermal agent was injected intravenously into 4T1 tumor-bearing mice. The tumor was subsequently irradiated at 808 nm with a power of 0.5 W cm^−2^ for 5 min, which resulted in a significant ablation of the mouse tumor, thus proving the efficacy of this organically synthesized nanomaterial. Green et al. studied the intratumoral injection of PEG-linked gold nanorods in subcutaneous Cal27 squamous cell carcinoma. In the tumor, when irradiated with 785 nm laser radiation with the power of 9.5 W cm^−2^ for 10 min, the treatment produced 100 percent tumor regression, a remarkable degree of success [193].

NIR-I, 700–900 nm has shown a promising record of success in the field of photothermal therapy. Recently, NIR-II window (NIR-II; 1000–1700 nm), either excitation or emission [194,195,196], is gaining the focus of the scientific community due to its positive results in vivo for deep tissue tumor ablation. It has been observed that NIR-II window exhibits high maximum permissible exposure (MPE) and a deeper depth of penetration than NIR-I. Cheng et al. studied the efficiency of an NIR-II window laser of 1275 nm on photothermal therapy over an NIR-I window laser of 808 nm. Polyethylene glycol stabilized copper sulfide nanoparticle (PEG-CuS NP) is used as a photothermal agent for this comparison study. Since this nanoparticle has the same absorption efficiency at 1275 nm and 808 nm, the nanoparticles were excited at these two wavelengths separately in vitro and in vivo for photothermal therapy study. The results demonstrated that the NIR-II window laser of 1275 nm is more efficient than the NIR-I window laser of 808 nm in raising the temperature in vitro and causing tumor destruction in vivo [197].

### 6.3. Photothermal Therapy in Human Clinical Trials

Photothermal therapy has shown promising results in in vitro and in vivo setups, as described above. However, its efficacy in human patients has been scarcely verified. Some clinical trials have been reported recently on the application of photothermal therapy on human cancer patients. The ClinicalTrials.gov cites only four clinical studies focusing on the application of a photothermal system; the undergoing human trials include NCT01679470, NCT00848042, NCT02680535, and NCT04240639. The nanomaterial involved in these trials/studies was Aurolase^®^, developed by Nanospectra Biosciences. This nanomaterial contains a core-shell structure of 150 nm. The core is dielectric and comprised of silica of 120 nm; however, the gold shell is 15 nm. Gold shell is known for a fascinating role in NIR light irradiation, leading to thermal ablation of tumors. Nanoparticle stability is achieved by the addition of polyethylene glycol (PEG). NCT01679470 was the first clinical trial involving patients with solid primary and metastatic cancer. The mode of administration of the above nanomaterial was intravenous. In this trial, bronchoscopy was used to administer NIR laser for thermal ablation of cancer. NCT00848042 was the second clinical trial involving patients with neck and head tumors. The mode of administration was selected to be intravenous. Thermal ablation was performed with 808 nm laser irradiation. NCT02680535 and NCT04240639 clinical trials involve patients with prostrate tumor. The study in NCT02680535 involves the focal ablation of prostate cancer by MRI/ultrasound-guided laser irradiation. NCT04240639 is an extension study of NCT02680535 [198,199].

## 7. Conclusions

In the above account, we have scrutinized and analyzed advancements from conventional thermal therapies to nanotechnology-based advanced photothermal therapies. Conventional thermal therapies, as shown above, include hyperthermia, diathermia, radiofrequency ablation, microwave ablation, and focused ultrasound ablation. The role of nanotechnology in thermal therapies and photothermal therapy has been highlighted based on recent studies in in vivo as well as in vitro settings. The reasons nanomaterials are increasingly used in thermal therapies are due to their small size, unique shape, optoelectronic properties, and ability to produce heat upon external source excitation. In addition, nanoparticles have dispersibility in biocompatible solvents and are largely compatible to biological systems. Detailed modes of action of magnetic and nonmagnetic nanoparticles have been elucidated in thermal and photothermal therapies in the above discussion. The various nanostructures that have shown greater efficacy in photothermal therapy include metallic nanoparticles, graphene nanostructure, carbon nanotubes, quantum dots, hybrid nanoparticles, and transition-metal-based nanosheets. Some nanostructures, such as transition metal dichalcogenides (TMD), have particularly attracted the attention of the scientific community worldwide. TMD also exhibits significantly strong absorption in the NIR region and spin orbit coupling, making it an ideal photoacoustic imaging contrast agent. Studies discussed above indicate that CoFeMn dichalcogenides nanosheets and some nanodots were capable of removing a tumor completely in in vivo conditions. These nanomaterials act as efficient photothermal agents in the ablation and control of tumors. The key factors that determine the PTT effects on solid tumors are mainly the penetration depth of irradiation into tumor tissue and the absorption of photothermal transduction agents (PTAs). Further, it was shown how the shape, size, and other unique characteristics of nanomaterials influence the desired photothermal effects. Photothermal therapy is showing a promising approach in human clinical trials. From this review, we can conclude that nanomaterial-mediated photothermal therapies can be a transformative development in the treatment and control of some cancers, particularly non-invasive tumors.

## Figures and Tables

**Table 1:** Potential nanomaterials for photothermal therapy.

Nanomaterials	Configuration	Wavelength	Power and Duration	Reference
**Metallic Nanostructures**				
Gold	Gold nanostar	980 nm	15 W cm^−2^, 5 min	[108]
Gold	PEG-gold nanostar	785 nm	1.1 W cm^−2^, 5 min	[108]
Copper	PEG-coated copper nanowires	808 nm	-	[111]
**Graphene nanostructures**				
Graphene oxide (GO)	Glycol functionalized graphene oxide (GO-PEG) nanosheet	808 nm	2 W cm^−2^	[132]
Graphene oxide (GO)	Gold-nanoparticle-specific aptamer–graphene oxide	808 nm	3 W cm^−2^, 5 min	[133]
**Carbon nanotubes**				
Single-walled carbon nanotubes (SWNT)	folate (FA)-functionalized SWNT	980 nm	0.5 to 1 W cm^−2^, 2 min	[148]
carbon nanotubes (CNT)	Oxidized CNT (O-CNT), and further functionalized it with PEG	808 nm	2 W cm^−2^, 10 min	[139]
**Quantum dots**				
CdSe quantum dot and CdTe quantum dots	CdSe, CdTe quantum dots	671 nm	0.16 W cm^−2^, 0–20 min	[154]
CdSe quantum dot and CdTe quantum dots	Mesoporous hollow NCQD captured carbon sphere	980 nm	1 W cm^−2^, 5 min	[155]
**Hybrid Nanoparticles**				
Au–Fe_3_O_4_ hybrid nanoparticles	Au-Fe_3_O_4_ hybrid nanoparticles	808 nm	0.5 W cm^−2^	[158]
Au–Pd hybrid nanoparticles	Au-Pd hybrid nanoparticles, functionalized with folic acid and chlorin e6	808 nm	1.5 W cm^−2^, 5 min	[159]
**Transition metal based nanosheets**				
CoFeMn dichalcogenides (CFMS) nanosheets	CoFeMn dichalcogenides (CFMS) nanosheets modified with polyvinyl pyrrolidone	808 nm	1 W cm^−2^, 5 on/off cycle, 10 min each cycle	[161]
MoS_2_ nano-sheets	PEGylated MoS_2_ nano-sheets	808 nm	Varying power, 5 min	[162]
MoS_2_ nanoflakes	PEG modified MoS_2_ nanoflakes (MoS_2_-PEG)	808 nm	2 W cm^−2^, 10 min	[163]
WS_2_ nanosheets	Ammonium ion intercalated 1T-WS_2_ ultrathin nanosheets (N-WS_2_)	808 nm	0.6 W cm^−2^, 10 min	[164]
**Polymer nanoparticles**				
polyaniline nanoparticles	F-127 functionalized polyaniline nanoparticles (F-PANP)	808 nm	0.5 W cm^−2^, 3 min	[170]
polyaniline nanoparticles	Folic-acid-functionalized lipid-coated polyaniline nanoparticles	808 nm	2 W cm^−2^, 5 min	[171]
polypyrrole	chitosan-polypyrrole-based nanocomposites	808 nm	2.0 W cm^−2^, 5 min	[172]
polypyrrole	DOX-loaded polypyrrole hollow nanocapsules (PPy HNCs)	980 nm	1.0 W cm^−2^	[173]
polydopamine	Polydopamine-coated cluster of iron oxide nanoparticles	808 nm	2 W cm^−2^, 10 min	[174]
poly-(3,4-ethylenedioxythiophene):poly(4-styrenesulfonate) (PEDOT:PSS)	polyethylene glycol (PEG)-coated PEDOT:PSS nanoparticles-based drug carrier	808 nm	0.15 W cm^−2^, 20 min	[175]

## Data Availability

Not applicable.
